# Cascade Control of Antagonistic VSA—An Engineering Control Approach to a Bioinspired Robot Actuator

**DOI:** 10.3389/fnbot.2019.00069

**Published:** 2019-09-04

**Authors:** Branko Lukić, Kosta Jovanović, Tomislav B. Šekara

**Affiliations:** School of Electrical Engineering, University of Belgrade, Belgrade, Serbia

**Keywords:** antagonistic actuator, tendon-driven actuators, variable stiffness actuators, bioinspired robotics, physical human–robot interaction, position–stiffness control

## Abstract

A cascade control structure for the simultaneous position and stiffness control of antagonistic tendon-driven variable stiffness actuators (VSAs) implemented in a laboratory setup is presented in the paper. Cascade control has the ability to accelerate, additionally stabilize, and reduce oscillations, which are all extremely important in systems such as a tendon-driven compliant actuators with elastic transmission. Inner-loop controllers are closed in terms of motor positions, and outer-loop controllers in terms of actuator position and estimated stiffness. The dominant dynamics of the system (position and stiffness), composed of the mechanical part and inner loops, are identified by a closed-loop auto-regressive with exogenous input (ARX) model. The outer-loop controllers are tuned on the basis of experimentally identified transfer functions of the system in several nominal operating points for different stiffness values. After the system is identified, a controller bank is generated in which a pair of actuator position and stiffness controllers correspond to a nominal operating point and covers the area surrounding the nominal point for which it is designed. The controllers used are integral-proportional differential (I-PD) and integral-proportional (I-P) controllers, which are a variation of the PID and PI controllers with dislocated proportional and derivative gains from a direct to feedback branch that result to no overshoot for even fast reference changes (i.e., step signal), which is essential for preventing tendon slackening (meeting the pulling constraint). Analytical formulas for controller tuning based on only one parameter, λ, are also presented. Since position and stiffness loops are decoupled, it is possible to change λ for both loops independently and adjust their performance separately according to the needs. Also, the controller structure secures the smooth response without overshooting step reference or step disturbance signal, which make practical implementation possible. After all the controllers were designed, the cascade control structure for simultaneous position and stiffness control was successfully evaluated in a laboratory setup. Thus, the presented control approach is simple to implement, but with a performance that ensures a pulling constraint for tendon-driven actuators as a foundation for bioinspired antagonistic VSAs.

## Introduction

Robotics has made major strides in recent decades. They began with the deployment of the first industrial robots in a known environment without humans in their immediate proximity. These robots had standard stiff actuators, while elastic deformation in their transmission system was deemed undesirable. The undesirable elasticity could induce oscillations, which engineers attempted to eliminate as early as the mechanical system design stage (Potkonjak, 1988). As industry and medicine developed, and society became more demanding, the need arose for robots and various types of electromechanical devices and drives, whose desired features are highlighted in application areas such as rehabilitation aids, exoskeletons, or service robots. New-generation robots are expected to operate in the immediate vicinity of humans or in collaboration with humans under dynamic conditions where the environment is unknown and changeable. To that end, a new generation of human-like (i.e., bioinspired) actuation is required with the first robotics applications in biologically inspired musculoskeletal humanoids (Diamond et al., 2012; Nakanishi et al., 2013). A number of actuation approaches that resemble properties of muscle system have been developed—tendon-driven and compliant drives that require both side pulling units (antagonistic actuators). For this to be feasible, safe interaction needs to be ensured in some way.

Progressively moving toward the next generation of robotic actuators, the active compliance and stiff actuator shortfalls are overcome with the development of passively compliant actuators. Compliant actuators are often designed as serial elastic actuators (SEAs) with constant actuator stiffness (Pratt and Williamson, 1995; Robinson et al., 1999), or as variable stiffness actuators (VSAs) that, in addition to the position, can control stiffness at the actuator output (Vanderborght et al., 2013; Grioli et al., 2015). Compliant actuators were developed in response to the need to ensure robot compliance and, consequently, safe interaction with the environment and primarily with humans. One of the compliant actuator advantages is that in the event of a collision with the environment, there is no need to wait for the controller to react. Instead, the impact energy is instantaneously absorbed and stored in the actuator's transmission system through deformation of elastic elements. This reduces the effects of interaction forces and ensures safe human–robot interaction. Compliant actuators are more energy efficient because they can utilize the stored energy, which is especially useful in the case of repetitive tasks. When compliant actuators release the stored energy, they exceed stiff actuator performance with regard to peak velocity (Lakatos et al., 2014).

Robots, in general, are designed for specific tasks and specific movements within those tasks. Rigid robots can simultaneously control position and stiffness only with additional feedback loops. The solution for this problem is in the biologically inspired actuator design approach, where actuation is based on the principles of antagonism (antagonistic controlled stiffness), copied from humans and animals and implemented in robots (Migliore et al., 2005; Koganezawa et al., 2006; Jovanovic et al., 2014). Therefore, antagonistic actuators are the bioinspired solution for VSAs, which should bring robot actuation closer to superior human actuation mastered through the evolution. This approach in actuator design improves the quality and variety of robotic movements. Results from Migliore et al. (2005) present loop control of antagonistic VSA, where achieved and commanded position and stiffness have a high level of correlation.

Observing the actuation of living creatures evolved through the centuries, contemporary robot joints are often tendon driven (Mizuuchi et al., 2002; Potkonjak et al., 2011b). The drives are relocated from the joints, and the joints are controlled by tendons wound on reels. The following are some of the implemented tendon-driven robots: the tendon-controlled humanoids Kenshiro, with 64 joint degrees of freedom (DOFs); Kengoro, with 114 joint DOFs without hands (Nakanishi et al., 2012; Asano et al., 2016, 2017); and ECCEROBOT (Wittmeier et al., 2013), which is a fully anthropomorphic, compliantly driven robot. Relocation of the actuators from the arms to the body of the robot reduces the inertia of the arms, which are then lighter and require less energy for control, but elastic elements cause more pronounced oscillations. Some of the challenges are to design an adequate control strategy for this type of actuator, taking into account its non-linearity originating from the condition for the system to have non-linear transmission so that stiffness can be controlled (Van Ham et al., 2009) and to avoid slacking through minimal pre-tensioning of the springs (Potkonjak et al., 2011a). In addition to VSA position and stiffness control in antagonistic actuators such as dynamic feedback control law for input–output decoupling and full state linearization (Palli et al., 2007), or the non-interacting static feedback linearization (Palli et al., 2008), both only validated in simulations, it is possible to apply concepts such as puller–follower (Potkonjak et al., 2011b), where the position and tension forces in the tendons are controlled instead of the VSA position and stiffness in order to preserve tension in tendons. Tendon-driven mechanisms can offer additional control flexibility by exploiting configurations with redundant non-linear elastic tendons, considering conditions under which the joint stiffness is adjustable (Kobayashi et al., 1998).

Newer research is focusing on deriving the control methods for simultaneous position and stiffness control such us feedback linearization for decoupled position and stiffness control with momentum-based collision detection (De Luca et al., 2009), impedance control with static decoupling (Wimbock et al., 2008), or the puller–follower concept where controllability issues are overcome by switching to force–position control (Potkonjak et al., 2011b). However, all abovementioned approaches miss experimental validation, which will make wide use of the presented research. Adaptive neural network control of tendon-driven mechanism is experimentally validated (Kobayashi and Ozawa, 2003), but it highlighted stability issues with the presence of the unmodeled dynamics.

This research was motivated by the wish to arrive at a simple, efficient, and robust control system, which can be applied regardless of the type of antagonistic VSA, based on the identification/estimation of the model of the system. The focus of this paper is on the antagonistic tendon-driven type of VSA. The implementation and testing of the proposed cascade control structure with the engineering control approach to an antagonistic actuator is the first step in controlling the broader class of VSAs. The objective is for the control structure to properly control the tendon-driven antagonistic actuator, with no prior knowledge about the model—only some actuator physical parameters that are easy to measure are considered known (i.e., dimensions of motors and actuator pulley radiuses), which will be discussed below. The motivation also traces to earlier activities and papers of the authors in the areas of modeling (Jovanovic et al., 2014) and control (Potkonjak et al., 2011a,b) of antagonistically driven robots. There is some research presenting the successful implemented simulations of position–stiffness control of antagonistic VSA: backstepping control implemented to an antagonistically driven finger with flexible tendons (Chalon and d'Andréa-Novel, 2014), or joint impedance controller with underlying tendon force control that was proposed in Chalon et al. (2011), in both cases, control structure is derived using the analytical model. No widely accepted practical implementation of these methods has been accomplished due to major dependence on the control model, such that the present paper constitutes an upgrade toward the implementation of simultaneous stiffness and position control. Issues in practical implementation for control of antagonistic actuators with the elastic transmission with cascade scheme were pointed out in Lukic et al. (2018). In this paper, the issues are resolved by closed-loop parameter identification and, consequently, the design of robust control scheme with guidelines for parameter tuning. Control structure with a decoupler enables independent control loops for actuator position and stiffness, where controller parameters in each loop are tuned with a variation of only one tuning parameter λ (in general, position and stiffness loop have different λ). Therefore, trade-off between performances and robustness is made by tuning λ. A more detailed explanation of controller tuning and its influence on performances will be presented in the section *Antagonistic VSA Cascade Position/Stiffness Control*.

In line with all that was previously stated, the presented research introduces a conventional engineering control approach to a bioinspired tendon-driven compliant antagonistic actuator as a concept of new widely accepted safe and efficient robot solution within a human-centered environment. The contribution of this paper is an experimentally validated approach for position–stiffness control of antagonistic VSA. The control is implemented without knowing the exact mathematical model of the actuator (i.e., parameters of non-linear spring characteristics, the transfer function of DC motors, gearbox efficiency coefficient, friction, etc.) but using widely accepted model identification tools for actuator system modeling. Thus, the approach can easily be applied to other types of antagonistic VSAs since the exact VSA parameters are rarely manufactured to fully match its mechanical design due to the complexity of their structure. The paper proposes a control scheme and procedure for controller tuning of antagonistic VSAs that is easy to implement while keeping tendons under tension to prevent slacking. The identified transfer functions present locally linear behavior of the system; thus, controllers are tuned to satisfy stability criteria for linear systems with a certain amount of robustness. The control design is simplified to the selection of one parameter for trading-off between performances and robustness, which shapes all the parameters in the introduced control structure.

In our approach, we presented a cascade control structure for position–stiffness control of antagonistic VSAs, where controller tuning is achieved based on an identified system dynamic. The cascade control structure gives better performance for reference tracking than the classical single-loop control system (Song et al., 2003). Our approach gives a simple procedure for control design through the tuning of free parameter λ. Some papers (Matausek and Sekara, 2011; Sekara et al., 2011; Boskovic et al., 2017) give insight into how controller parameters are tuned as a function of the parameter λ. Our approach requires no prior knowledge of the system model nor higher-order derivatives, and it is easy to implement to a real setup.

The decentralized trajectory tracking (Della Santina et al., 2017; Angelini et al., 2018) is a method where the feedforward component is learned in an iterative procedure to have a good trajectory tracking performance while minimizing the influence of the feedback component. Feedforward control does not affect robot stiffness; thus, natural robot softness is preserved. Shortcomings of this method, compared to ours, are that for every new trajectory, it is necessary to repeat the learning method, which can take a lot of time.

The elastic structure preserving (ESP) and ESP+ control approaches (Keppler et al., 2018) change plant dynamics less than feedback linearization-based control and aim to minimize the dynamic shaping and preserve the elastic structure of the system. The limitation of this approach is that the model-based control law requires higher-order derivatives of link positions, while in our case, a control law is model free and exploits only information of actuators and link positions.

Section Antagonistic VSA Drive: Prototype and Modeling of the paper describes the general structure, mathematical model, and physical implementation of the laboratory setup of the bioinspired antagonistic VSA, as well as the way in which the system was identified. The cascade control structure and controller tuning are discussed in the section *Antagonistic VSA Cascade Position/Stiffness Control*. The section *Experiment Results* contains the experimental results, and the section *Conclusions* summarizes the conclusions and directions of future research.

## Antagonistic VSA Drive: Prototype and Modeling

**Figure 1:**
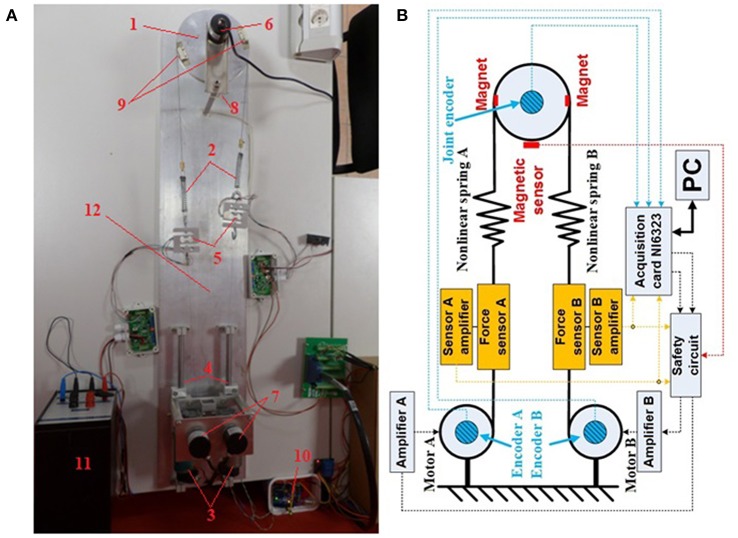
**(A)** Laboratory hardware configuration of the tendon-driven antagonistic variable stiffness actuator: (1) actuator link; (2) non-linear compression springs; (3) DC motors; (4) sliding mechanism for pre-tensioning; (5) force sensors; (6) actuator incremental encoder; (7) gearbox shaft incremental encoders; (8) magnetic sensor; (9) magnets; (10) safety circuit; (11) power source; and (12) pedestal. **(B)** Block structure of the tendon-driven variable stiffness antagonistic actuator. Blue denotes encoders, red magnets and the magnetic sensor, and orange force sensors. Force (dashed orange lines) and actuator and motor position (dashed blue lines) measurements are interfaced with the computer via the NI-PCIe6323 acquisition card. Computed control signals are forwarded via analog outputs of the acquisition card to the safety module. The safety module operates independently and acquires force sensor data and end-position magnetic sensor data (dashed red line).

Figure 1A shows the mechanical configuration of an antagonistic VSA developed in the ETF Robotics Laboratory of the University of Belgrade. The laboratory setup comprises (1) a link; (2) a non-linear compression springs with a quadratic characteristic; (3) two Dunker GR42X25 DC motors with Dunker SG45 gearbox, ratio 15:1; (4) a sliding mechanism for pre-tensioning; (5) two TAL501L force sensors made by HT Sensor Technology, which measure tendon tension; (6) an ISC3806-003E-1000BZ3-5-24F incremental rotary encoder for actuator position tracking; and (7) two DHC40M6-2000 incremental rotary encoders for position tracking at the gearbox outlet; (8) an end-position magnetic sensor; (9) magnets; (10) a safety circuit; (11) a power sources; and (12) a pedestal.

The structure of the actuator is attached to a plate acting as a pedestal of the apparatus. The antagonistic drive itself is positioned on sliders used to adjust spring pretension. This also facilitates spring replacement and pre-tensioning, if springs of different lengths and characteristics are to be used. The apparatus was sized such that the link radius at the gearbox outlet is *r*_*m*_ = 7.5 *mm* and the actuator radius *r*_*j*_ = 75 *mm*. Signals are acquired by a National Instruments NI-PCIe6323 acquisition card with analog outputs for motor control.

Figure 1B is a block structure of the antagonistic VSA. Force (dashed orange lines) and actuator and motor position (dashed blue lines) measurements are interfaced with the computer via the NI-PCIe6323 acquisition card. Computed control signals are forwarded via analog outputs of the acquisition card to the safety module. The safety module operates independently and acquires force sensor data and end-position magnetic sensor data (dashed red line). If all signals are within prescribed boundaries, the control signals are forwarded to the motors, and if the actuator position or tension exceeds the permissible level, the motors immediately shut down to prevent damage to the apparatus. The control structure was implemented in a Matlab/Simulink Real-Time environment.

## Mathematical Model of Antagonistic VSA

The tendon-driven antagonistic VSA operates in such a way that motor rotation around outlet shaft groves causes the tendons to wind/unwind and, thus, compress/decompress the springs. Simultaneous compression increases actuator stiffness, while winding of one tendon and unwinding of another changes the position. In order to control antagonistic actuator stiffness, the stiffness in the transmission mechanism (in this case spring) between the motor and the link needs to be non-linear, with a monotonously incremental force relative to extension (Van Ham et al., 2009). The pair of springs used in the apparatus were custom-made. The requirements were that they be identical and have a monotonous growing force/elongation characteristic.

Spring elongations Δ*l*_*A*_ and Δ*l*_*B*_ of the antagonistic actuator are defined by:

(1) $$\begin{array}{r} \Delta l_{A}=r_{m}\theta_{A}-r_{j}q \end{array}$$

(2) $$\begin{array}{r} \Delta l_{B}=r_{m}\theta_{B}+r_{j}q \end{array}$$

where the positions of the actuator, motor A and motor B are *q*; θ_*A*_, and θ_*B*_, respectively.

Force/elongation characteristic can be represented in a polynomial form as:

(3) $$\begin{array}{r} F_{A}=\sum_{i=0}^{n}k_{i}\Delta l_{A}^{i} \end{array}$$

(4) $$\begin{array}{r} F_{B}=\sum_{i=0}^{n}k_{i}\Delta l_{B}^{i} \end{array}$$

where *F*_*A*_ and *F*_*B*_ denote forces that compress the springs, where *k*_*i*_ for *i* = 1…*n*, polynomial coefficients, and *n* is the order of the polynomial.

**Figure 2:**
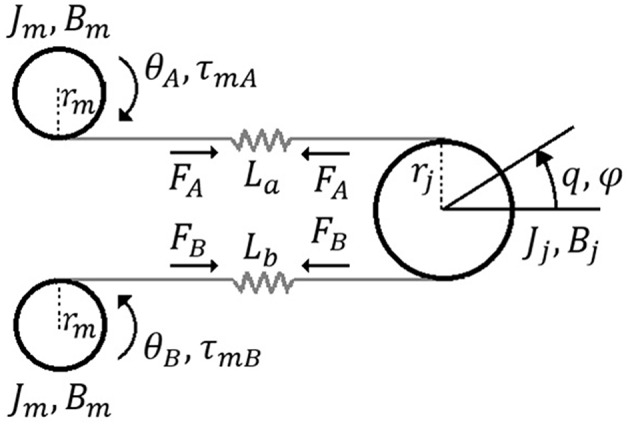
Schematics of antagonistic tendon-driven VSA.

The schematics of the variable stiffness antagonistic actuator is depicted in Figure 2, while a model that describes actuator dynamics and the dynamics of motors A and B is given in Equations (5) to (7):

(5) $$\begin{array}{r} J_{j}\ddot{q}+B_{j}\dot{q}=\varphi \left(\theta_{A},\theta_{B},q\right) \end{array}$$

(6) $$\begin{array}{r} J_{m}{\ddot{\theta }}_{A}+B_{m}{\dot{\theta }}_{A}+\phi \left(\theta_{A},q\right)=\;\tau_{mA} \end{array}$$

(7) $$\begin{array}{r} J_{m}{\ddot{\theta }}_{B}+B_{m}{\dot{\theta }}_{B}+\psi \left(\theta_{B},q\right)=\;\tau_{mB} \end{array}$$

where *J*_*j*_ and *J*_*m*_ are actuator and motor inertia, respectively. *B*_*j*_ and *B*_*m*_ symbolize the equivalent viscous friction in the actuator and motors, respectively. The torques of motors A and B are denoted by τ_*mA*_ and τ_*mB*_, respectively, and can be represented as:

(8) $$\begin{array}{r} \tau_{mA}=\text{μ}N\frac{K_{mu}}{Ls\;+\;R}U_{A} \end{array}$$

(9) $$\begin{array}{r} \tau_{mB}=\text{μ}N\frac{K_{mu}}{Ls\;+\;R}U_{B} \end{array}$$

where *L* and *R* are induction and resistance of a motor, respectively. *K*_*mu*_; *N*, and μ symbolizes the motor torque constant, gearbox reduction ration, and gearbox coefficient of efficiency, respectively. *U*_*A*_ and *U*_*B*_ are voltage inputs on motor A and motor B, respectively. The *s* is Laplace transformation operator.

The equivalent actuator torque and parts of the actuator torques originating from the elastic springs connected with motor A and motor B are φ; ϕ, and ψ, respectively (shown in Equations 10–13):

(10) $$\begin{array}{r} \phi \left(\theta_{A},q\right)=r_{m}F_{A} \end{array}$$

(11) $$\begin{array}{r} \psi \left(\theta_{B},q\right)=r_{m}F_{B} \end{array}$$

(12) $$\begin{array}{r} \varphi \left(\theta_{A},\theta_{B},q\right)=\frac{r_{j}}{r_{m}}\left(\phi \left(\theta_{A},q\right)-\;\psi \left(\theta_{B},q\right)\;\right) \end{array}$$

by combining Equations (10) to (12), Equation (13) for actuator torque follows:

(13) $$\begin{array}{r} \varphi \left(\theta_{A},\theta_{B},q\right)=r_{j}\left(F_{A}-F_{B}\right) \end{array}$$

Actuator's stiffness *S* is defined by Equation (14).

(14) $$\begin{array}{r} S=-\frac{\partial \varphi \left(\theta_{A},\theta_{B},q\right)}{\partial q} \end{array}$$

The equilibrium position of the actuator *q*_*e*_ for the symmetrical system shown in Figure 1, based on known geometric relations and symmetry, is:

(15) $$\begin{array}{r} q_{e}=\frac{r_{m}\left(\theta_{A}-\theta_{B}\right)}{2r_{j}} \end{array}$$

The expression for actuator stiffness is derived by combining Equations (3) and (4) with Equations (13) to (15):

(16) $$\begin{array}{r} S=2r_{j}^{2}\sum_{i=1}^{n}\frac{i}{2^{i-1}}k_{i}r_{m}^{i-1}{\left(\theta_{A}+\theta_{B}\right)}^{i-1} \end{array}$$

## Parameter Estimation

In order to make the approach versatile and applicable to a different kind of antagonistic VSAs, several main actuator characteristics have to be estimated. Initially, each individual drives and gearing have to be identified as a core of the internal loop. Elastic transmission elements determine the actuator dynamics, and consequently, they have to be well-estimated for the control purposes as well as for the evaluation of the actuator performances. Finally, for the sake of reliable and robust position and stiffness control, outer loops that comprise inner loops, elastic transmission, and actuator mechanical design have to be identified.

Fast system dynamics is determined by the electric drive and its gearbox. The goal of the control approach is to accurately and promptly control antagonistic drive positions so the outer position and control loops are preserved. In order to tune the inner loop, the parameters of the assumed geared motor transfer function *G*_*m*_(*s*) were identified. The transfer function was assumed to be a second-order transfer function without time delay, where voltage is the input and motor position the output. The parameters were estimated by first estimating those of the motor speed transfer function, without payload, described as a first-order transfer function without time delay, which is a common approximation in engineering. In general, velocity/voltage transfer function of the motor gearbox set is a second-order transfer function with two real poles on the left side of the complex plane, where one pole is determined by electric characteristics and the second pole is determined by mechanical characteristics. The mechanical component of the system is significantly slower than electrical; thus, its dynamic behavior is dominant and transfer function can be estimated as a first-order transfer function (Leonhard, 2001; Ogata, 2009). Gain *K*_*m*_ and time constant *T*_*m*_ were estimated from the input specified as a step signal and from the motors' velocity step response. Then, an integrator was added to the resulting transfer function because the position is the integral of speed. In accordance with the adopted first-order transfer function, gain *K*_*m*_ is estimated as the ratio between the input voltage and output velocity, while time constant *T*_*m*_ presents the time required to motor reach 63.21% of the steady-state value. Identification experiments were executed for several input amplitudes. Parameters *K*_*m*_ and *T*_*m*_ were estimated as mean values of all experimental results, while there were no significant deviations from the mean values. Hence, the motor transfer function *G*_*m*_(*s*) with estimated parameters is:

(17) $$\begin{array}{r} G_{m}\left(s\right)=\frac{K_{m}}{s\left(T_{m}s+1\right)} \end{array}$$

where *K*_*m*_ = 2.86 is the estimated gain factor and *T*_*m*_ = 0.22 is the estimated time constant.

**Figure 3:**
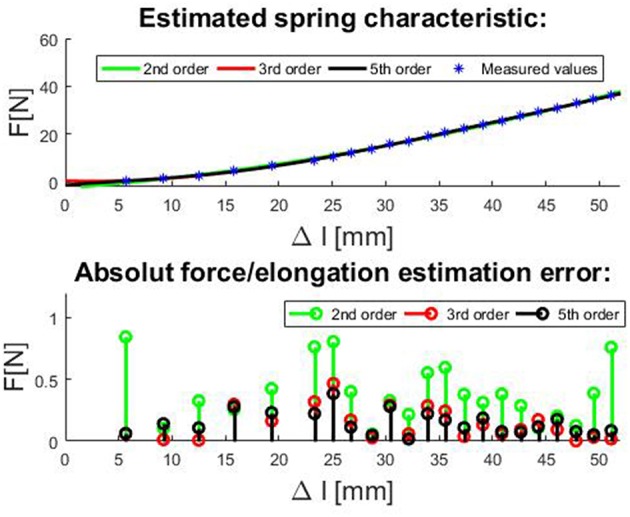
Top plot: The fitting curves for compression spring with non-linear force/elongation characteristic for 2nd, 3rd, and 5th order polynomial model satisfying the least square error and. Bottom plot: Estimation errors of spring force/elongation characteristic for 2nd, 3rd, and 5th order polynomial.

Actuator dynamics and its stiffness are determined by elastic elements used in the transmission. In order to estimate actuator stiffness according to (16), spring parameters need to be estimated as well. If actuator elastic elements/springs are changed, spring parameters must be estimated again. To accurately determine the spring coefficients, the spring characteristic was captured by measuring forces and spring elongations at different loads. The fits of the spring function for 2nd-, 3rd-, and 5th-order polynomial satisfying the least square error are shown on the top plot, while estimation errors are shown on the bottom plot in Figure 3. As expected, with polynomial order increase, estimation error decreases. From the observation of the measurement and polynomial approximations, the 2nd-order produces error <0.85 N and the higher polynomial order do not decrease error significantly. For controlling the tendon-driven actuators, there is always some minimal tension required in tendons (non-linear springs); thus, the amplitude of errors will be relatively small in comparison with the amplitudes of working forces (Potkonjak et al., 2011b). The 2nd-order polynomial model presents the compromise between the accuracy on the one side and its easy implementation on the other side. In cases when this error amplitude is not negligible, the higher-order model must be used. The resulting coefficients are *k*_0_ = −2.498 *N, k*_1_ = 331.49*N*/*m*, and $k_{2}=\;8543.8N/m^{2}$.

**Figure 4:**
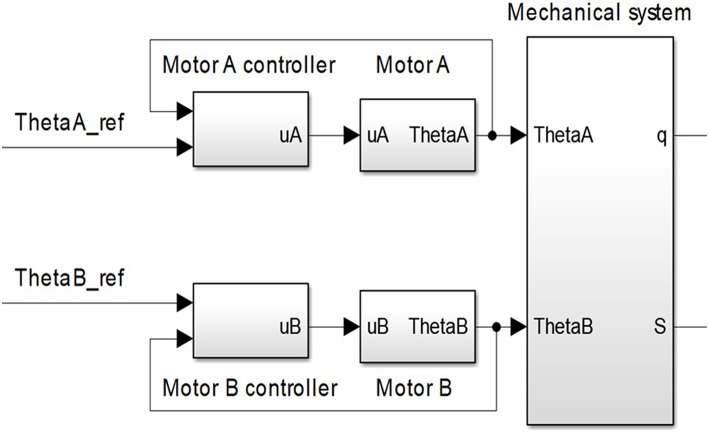
Part of the control system whose dominant dynamic needs to be identified. This part of the control structure consists of system mechanics and inner motor position loops.

**Figure 5:**
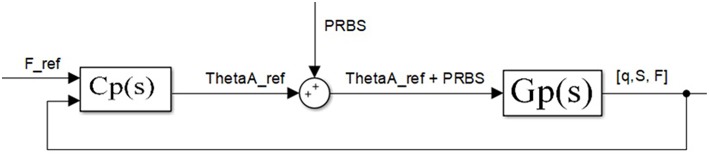
Tendon tension force closed-loop. Block diagram for experimental estimation of actuator outer loops—(actuator position and stiffness). Identifying dominant dynamics represented as a transfer function for the position—*G*_*q*_ (*s*) and stiffness *G*_*S*_ (*s*) are grouped in *G*_*p*_ (*s*).

Finally, actuator performances depend on its electromechanical design as a whole. Figure 4 shows the structure of the system whose dominant dynamics is identified. The system is composed of the mechanical part of the antagonistic VSA and motor position inner loops. The system inputs are reference motor positions, and the outputs are actuator's stiffness and position. The transfer functions of real systems, which are continuous in nature, based on periodic discrete measured data (from computer or microcontroller), can readily be represented by a discrete equivalent such as the ARX model (autoregressive model with exogenous inputs) (Jansson, 2003). Identification was undertaken in a closed loop as described in Mataušek and Šekara (2014) and Hjalmarsson (2005), and it is shown in Figure 5. Close loop identification is required to reduce the effect of sensor noise and avoid output drift (Skogestad and Postlethwaite, 2001), which cannot be achieved through the open-loop identification.

Figure 5 depicts the force control loop used only for the identification of outer loop transfer functions—actuator position [*G*_*q*_(*s*)] and stiffness [*G*_*S*_(*s*)]. The control loop for identification consists of controller *C*_*p*_(*s*) and transfer function *G*_*p*_(*s*) that include both actuator position and stiffness transfer function: *G*_*q*_(*s*) and *G*_*S*_(*s*). Transfer function *G*_*p*_(*s*) includes the mechanical design of the antagonistic actuator with elastic transmission elements and inner loops as shown in Figure 4. Controller *C*_*p*_(*s*) controls the tendon tension force in the outer loop around a nominal point. For identification purposes, controller *C*_*p*_(*s*) in the outer loop does not need to be optimally tuned but stable. In general, the feedback need not to be same as the output variable, which is being identified, it only needs to be stable in order to avoid output drift and keep the system around a nominal setpoint. Also, closed-loop identification is carried out to reduce the influence of sensor noise and therefore improve identification. Based on the inputs and the outputs of the system *G*_*p*_(*s*), dominant dynamics of the actuator position and stiffness is identified.

The system shown in Figure 4 has two inputs: motor positions θ_*A*_ and θ_*B*_, and two outputs: actuator position and stiffness *q* and *S*. Given that the system is symmetrical and that both inputs contribute equally to the output, identification is undertaken with respect to only one motor, while the other is kept still. Without loss of generality, it is deemed that θ_*B*_ = 0.

Since the system is non-linear, it was identified in the vicinity of several operating points, for tension forces of 10, 20, 30, and 40 N, which correspond to the estimated stiffnesses of 8.24, 10.55, 12.43, and 14.06 Nm/rad, respectively. The stiffness range is limited by the properties of the actuator design and compression springs, which are fully compressed at 50 N. However, the proposed control approach is invariant to the stiffness range.

While the outer loop keeps the system at a nominal tension force, a low-amplitude PRBS (pseudorandom binary sequence) signal is added to the input-desired motor position, just enough for its effect to be visible at the output and distinguishable from sensor noise (Polóni et al., 2008). The PRBS with a sampling period of 1 ms was applied. The one sequence of the PRBS signal consists of binary signals generated under defined rules (rules for generating the PRBS signal refer to the shift registry and polynomial rules based on the signal length–number of samples within one period) (MacWilliams and Sloane, 1976), with the property of constant amplitude spectrum that excites the system on all frequency equally.

Based on the measured input of the system *G*_*p*_(*s*) (motor A reference position of _θ_*A*_*ref*_) and output (actuator position, *q*; stiffness, *S*; and tendon tension force, *F*), the ARX model was used to identify the dominant dynamics for actuator position and stiffness at each of the nominal values. The dominant dynamics is the lowest-order polynomial transfer function whose response adequately mimics the real response of the system. Table 1 shows the estimated transfer functions of the system for position *G*_*q*_(*s*) and stiffness *G*_*S*_(*s*) of the antagonistic actuator at various nominal points obtained through identification, where *G*_*q*_(*s*) and *G*_*S*_(*s*) are grouped in *G*_*p*_(*s*) from Figure 5.

**Table 1:** Estimated transfer functions for actuator position *G*_*q*_ (*s*) and stiffness *G*_*S*_ (*s*) in various nominal points.

**Nominal**	**1st** **order estimation**		**3rd** **order estimation**	
	**G_q_(s)**	**G_S_(s)**	**G_q_(s)**	**G_S_(s)**
*F* = 10 N	$\frac{0.0498}{0.64s+1}$	$\frac{1.17}{0.57s+1\;}$	$\frac{0.0498}{\left(0.64s+1\right)\left(7.329{*10}^{-5}\;s^{2}\;+\;0.05747\;s\;+\;1\right)}$	$\frac{1.17}{\left(0.57s+1\right)\left(2.3{*10}^{-5}s^{2}+1.6{*10}^{-3}s+1\right)\;}$
*F* = 20 N	$\frac{0.0484}{0.78s+1}$	$\frac{2.826}{1.19s+1\;}$	$\frac{0.0484}{\left(0.78s+1\right)\left(4.919{*10}^{-5}\;s^{2}+\;0.04688\;s\;+\;1\right)}$	$\frac{2.826}{\left(1.19s+1\right)\left(2.1{*10}^{-5}s^{2}+2.2{*10}^{-3}s+1\right)\;}$
*F* = 30 N	$\frac{0.0495}{1.58s+1}$	$\frac{1.821}{0.62s+1}$	$\frac{0.0495}{\left(1.58s+1\right)\left(3.475{*10}^{-5}\;s^{2}\;+\;0.03309\;s\;+\;1\right)}$	$\frac{1.821}{\left(0.62s+1\right)\left(1.3{*10}^{-5}s^{2}+4.2{*10}^{-3}s+1\right)}$
*F* = 40 N	$\frac{0.0491}{2.98s+1\;}\;$	$\frac{1.47}{0.43s+1}$	$\frac{0.0491}{\left(2.98s+1\right)\left(2.2*10^{-6}s^{2}+1.07*10^{-2}s+1\right)}$	$\frac{1.471}{\left(0.43s+1\right)\left(7.7*10^{-6}s^{2}+7.4*10^{-4}s+1\right)}$

Results are divided into two groups: first- and third-order estimations of the transfer function. All estimated function of the third order has one real pole and one pair of conjugated complex poles. A pair of conjugated complex poles produces some oscillatory dynamics, but the real pole is at much lower frequencies; thus, this dynamics is dominant and system transfer function can be approximated as the first-order transfer function. The predominance over the oscillatory dynamics could be explained by the existence of the inner loop controller, which shapes on overall input/output dynamics.

## Antagonistic VSA Cascade Position/Stiffness Control

**Figure 6:**
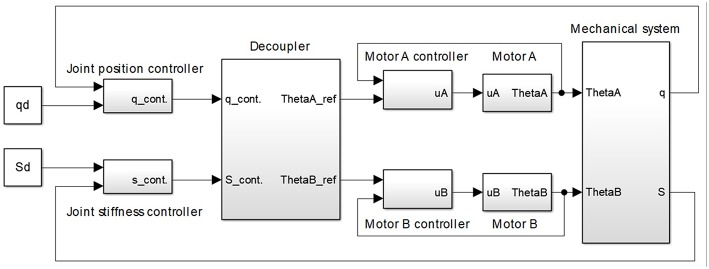
Cascade control structure for simultaneous position and stiffness control of antagonistic tendon-driven actuator. The control structure comprises: (1) a mechanical system with the antagonistic drive; (2) two motor-position inner loops; (3) actuator-position outer loops; (4) actuator-stiffness outer loops; and (5) a static decoupler that recalculates reference motor positions for the desired actuator position and stiffness.

The control structure is realized as a cascade control structure for simultaneous position and stiffness control, which is shown in Figure 6. Compared to a single feedback loop and having in mind that the inner and outer loops rely on the same control elements (DC motors), the cascade structure contributes to better performance of the system because the inner loop is designed to act locally and to always be faster than the outer loop (Skogestad and Postlethwaite, 2001). Furthermore, due to its faster dynamics, the inner loop adjusts DC motor behavior to tackle non-linearities (including friction) ahead of the outer loop, thereby minimizing negative effects of the tendon slackening, which is a crucial control issue in tendon-driven actuators (Lukic et al., 2018). The cascade structure removes a disturbance more effectively, thus achieving a faster response of the entire system and reducing oscillations, all of which are of major importance for tendon-driven robots to enable practical implementation. Thus, since the inner loop makes more rapid adjustments, the outer loop can be tuned more conservatively (Song et al., 2003). This is of particular importance since theoretical control approaches require smooth position and stiffness generation to the high-order derivatives.

The cascade control structure shown in Figure 6 comprises (1) a mechanical system with the antagonistic drive; (2) two motor position inner loops; (3) actuator position outer loop; (4) actuator stiffness outer loop; and (5) a static decoupler that computes reference motor positions for the desired actuator position and stiffness.

All controllers used in the inner and outer loops are integral-proportional differential (I-PD) or integral-proportional (I-P) controllers, with a filter (which filters out the sensor noise and ensures causality of the controller transfer function) (Åström and Hägglund, 2006; Shamsuzzoha and Lee, 2007). Introduced I-PD/I-P controllers facilitate appropriate parameter tuning, such that the performance of the closed-loop systems is satisfactory in terms of bandwidth, trajectory tracking, robustness, and disturbance rejection. Note that the bandwidth of the system is limited, and system response cannot be accelerated more than the mechanical properties allow.

Static decoupler *D* (Figure 6) reproduces the actuator position and stiffness at the output of the outer loop controllers *q*_*cont*_ and *S*_*cont*_ in reference motor positions θ_*A*_*ref*__ and θ_*B*_*ref*__. It is based on the estimated relations for the position (15) and second-order polynomial for stiffness (16) of the actuator in the state of equilibrium.

(18) $$\theta_{A_{ref}}=\frac{r_{j}}{r_{m}}q_{cont}+\frac{S_{cont}-2k_{1}r_{j}^{2}}{4k_{2}r_{j}^{2}r_{m}}$$

(19) $$\theta_{B_{ref}}=\frac{-r_{j}}{r_{m}}q_{cont}+\frac{S_{cont}-2k_{1}r_{j}^{2}}{4k_{2}r_{j}^{2}r_{m}}$$

Equations (17) and (18) can be written in matrix form as:

(20) $$\left[\begin{array}{c} \theta_{A_{ref}}\\ \theta_{B_{ref}} \end{array}\right]=D\left[\begin{array}{c} \begin{array}{c} q_{cont}\\ S_{cont} \end{array}\\ 1 \end{array}\right]$$

(21) $$D=\left[\begin{array}{ccc} \frac{r_{j}}{r_{m}}\; & \frac{1}{4r_{j}^{2}r_{m}k_{2}} & \frac{-k_{1}}{2k_{2}r_{m}}\\ \frac{-r_{j}}{r_{m}} & \frac{1}{4r_{j}^{2}r_{m}k_{2}} & \frac{-k_{1}}{2k_{2}r_{m}} \end{array}\right]$$

where matrix *D* is the system decoupler and $2k_{1}r_{j}^{2}$ is the minimum actuator stiffness derived from the estimated spring model under the pulling constraint.

In cases when stiffness is presented as higher-order polynomic or a complex non-linear equation (Catalano et al., 2011), it is not possible to present an analytical solution for decoupler function. Thus, the mapping between motor positions and actuator stiffness can be achieved with a lookup table or using some computing tool such as a neural network (Lukic et al., 2016).

The following part of this section introduces guidelines for inner and outer loop control design using complementary sensitivity function shaping. Following the control approach presented in Matausek and Sekara (2011), Sekara et al. (2011) and Boskovic et al. (2017), dynamic behaviors of the closed-loop systems are directly affected by the parameter λ as an only tuning parameter. Therefore, complementary sensitivity function shaping enables easy control design of the controller, its easy adaptation to the changes in the system, and its desired behavior by changing parameter λ. Lower values of λ mean faster closed-loop dynamics, but higher gains and, therefore, higher amplitude peaks in the control signal, and lower λ will provide slower system dynamics with longer but lower amplitude control signals. Some papers (Matausek and Sekara, 2011; Sekara et al., 2011; Boskovic et al., 2017) give insight into how controller parameters are tuned as a function of the parameter λ and its influence on the closed-loop dynamics. From these papers, rules for controller parameter tuning and closed-loop dynamics behavior shaping even for higher-order systems with parameter λ can be extracted. However, for the purpose of this paper, the only dynamical behavior of the first- and the second-order systems is required. The procedure for the derivation of controller parameters is given in Appendix A.

## Tuning of Inner Loop Controllers

This section introduces guidelines for tuning of the inner control loop–motor position control. Taking the parameters of the transfer function from Wittmeier et al. (2013), a robust I-PD controller was designed to reject disturbances and minimize the integral of the absolute error according to existing analytical formulas for several typical second-order systems, introduced and mastered by the third author (Matausek and Sekara, 2011; Sekara et al., 2011; Boskovic et al., 2017). Table 2 shows the controller parameters as functions of system (motor) parameters and tuning parameter λ, whose tuning affects system performance and robustness.

**Table 2:** I-PD and I-P controller analytical formulas for identified motor transfer function and first-order transfer function without time delay (*G*_*S*_(*s*) and *G*_*q*_(*s*)).

**Process**	**G_m_ (s)**	**G_S_ (s)/G_q_(s)**
Transfer function form	$\frac{K_{m}}{s\left(T_{m}s+1\right)}$	$\frac{K}{\left(Ts+1\right)}$
*k*_*p*_	$\frac{4T_{m}^{2}}{K_{m}\lambda^{2}\left(4T_{m}-\lambda \right)}$	$\frac{2}{K\lambda }$
*k*_*i*_	$\frac{T_{m}^{2}}{K_{m}\lambda^{3}\left(4T_{m}-\lambda \right)}$	$\frac{T}{K\lambda^{2}\;}$
*k*_*d*_	$\frac{6T_{m}^{2}-\lambda \left(4T_{m}-\lambda \right)}{K_{m}\lambda \left(4T_{m}-\lambda \right)}$	–
*k*_*r*_	*bk*_*p*_; *b* ∈ [0 − 1]	
*t*_*f*_	$\frac{T_{m}\lambda }{4T_{m}-\lambda }$	–

The controller was designed to satisfy the desired sensitivity function *ms*_*m*_, defined as $ms_{m}=\;\underset{\omega }{max}\left|\frac{1}{1+G_{m}\left(j\omega \right)C_{m}\left(j\omega \right)}\right|$, where *C*_*m*_(*jω*) is the controller that regulates the operation of the motor. The actuator range used in the experiments is 180 *deg*, which corresponds to 10 times wider motor range according to joint/motor pulley radius [see Equation (15)]. Another constraint in the experiments is the stiffness range (8–15 Nm/rad). The constraints are caused by the mechanical design of the actuator. Maximal disturbance introduced as an additional spring tension in robustness testing is 50 N, which corresponds to 0.375 Nm torque disturbance at the gearbox shaft. The value *ms*_*m*_ = 1.4 was determined experimentally; in such a way that it is a good trade-off between trajectory tracking performance and robustness. The desired value of *ms*_*m*_ for motor transfer function identification is achieved at λ = 0.0854. The controller parameter *k*_*r*_ can be tuned in the (0 − *k*_*p*_) range, and the value of parameter *b* is in the (0–1) range. If *b* is higher, when the reference changes, the system will respond more quickly but the overshoot will also be larger. Without loss of performance in terms of disturbance rejection and robustness, *b* = 0 and, hence, *k*_*r*_ = 0 is selected. Rejection of the overshoot is an essential requirement in the inner loop, which should prevent oscillation in compliant tendon-driven actuation. Since the system is symmetrical, both motors have the same controllers.

## Tuning of Outer Loop Controllers

This section introduces guidelines for tuning of the outer control loops–actuator position and stiffness control. For transfer functions of the first and third orders for position and stiffness given in Table 1, in each case, the dominant behavior is well-defined with its first-order transfer function; thus, the dominant dynamics can be represented as a first-order transfer function without time delay, with static gain *K* and time constant *T*.

Given that the transfer functions were identified as first-order transfer functions without time delay, the outer loop controllers were tuned like I-P controllers according to Boskovic et al. (2017). The I-PD/I-P controllers are variations of the PID/PI controllers, where the proportional and derivative gains from the direct branch are dislocated to the feedback branch, and it will reflect to no overshoot even for fast reference changes (i.e., step signal); thus, it will keep tendons under tension and prevent the slacking. Here, λ is again a tuning parameter that can be used to tune the response rate. The advantage of these controllers is that by tuning just one parameter, it is possible to make a balance between performance and robustness. The λ is a monotonic parameter, which means that a lower value always means a lower closed-loop system time constant and, therefore, faster response, but less robustness. For higher values of λ, results are opposite. Another benefit of centralized controller tuning is the avoidance of actuator saturation, which could deteriorate pulling constraint and performances of a tendon-driven system, especially if it comprises elasticity. To that end, a decrease in λ leads toward actuator limits and uses of full actuator range. Online changing of λ allows the implementation of gain scheduling depending on desired performances and response rate for different operating points. If the identification process reveals a more complex dominant dynamic, with multiple poles, the analytical formulas for the controller parameters can be derived as described in Matausek and Sekara (2011). Table 2 shows the analytical formulas for I-P controller tuning. To achieve a satisfactory response rate without overshoot, λ = 0.2 and *b* = 0 were adopted for all position and stiffness controllers and present initial tuning.

Given that the decoupler introduces a gain between the outer loop controller output and the reference positions of the motor, in order to retain the performance of the designed controllers, gains *k*_*p*_; *k*_*i*_, and *k*_*r*_ need to be divided by the gains introduced by the decoupler. According to the decoupler values from (20), actuator position gains should be divided by $\frac{r_{j}}{r_{m}}$ and the actuator stiffness controller gains by $\frac{1}{4r_{j}^{2}r_{m}k_{2}}$.

In the case of non-linear systems, due to different dynamics in different operating points, controllers can be designed for a linearized model, for each of the operating points of a set, thus forming a controller bank or set. The set of controllers can be used to control the system in such a way that each of the controllers would be tasked with a part of the operating range, for example, controller 1 would cover stiffness from *S*_1_ to *S*_2_, controller 2 from *S*_2_ to *S*_3_, controller 3 from *S*_3_ to *S*_4_, and so on. In this research, bumpless switching between controllers is used (the integral component takes over the control value while switching between two different controllers within the controller bank). Another application is to use a certain type of gain scheduling, where controller gains are computed as a linear combination of the two nearest controllers, depending on the nominal for which they were designed and on the desired value.

It is of most importance to ensure a pulling constraint and avoid tendon slacking. Therefore, outer loops could be easily prioritized to ensure pulling constraint by making position control slower than stiffness control, which guarantees no slacking and, thus, allows validation on the laboratory setup. This can be achieved by adjusting the parameter λ separately for each of the outer loops, since the response time is proportional to the parameter λ for each loop individually (Sekara et al., 2011). Thus, this parameter dictates the response dynamics by trading off between the robustness and performance.

## Experiment Results

Performances of controllers are verified in two experiments. The first experiment is position and stiffness tracking while their references are changing separately. In this scenario, actuator stiffness is estimated based on (13). In the second experiment, actuator stiffness/compliance control is validated by tendon force measurements, and therefore, stiffness is estimated directly from actuator torque and position measurement (12).

**Figure 7:**
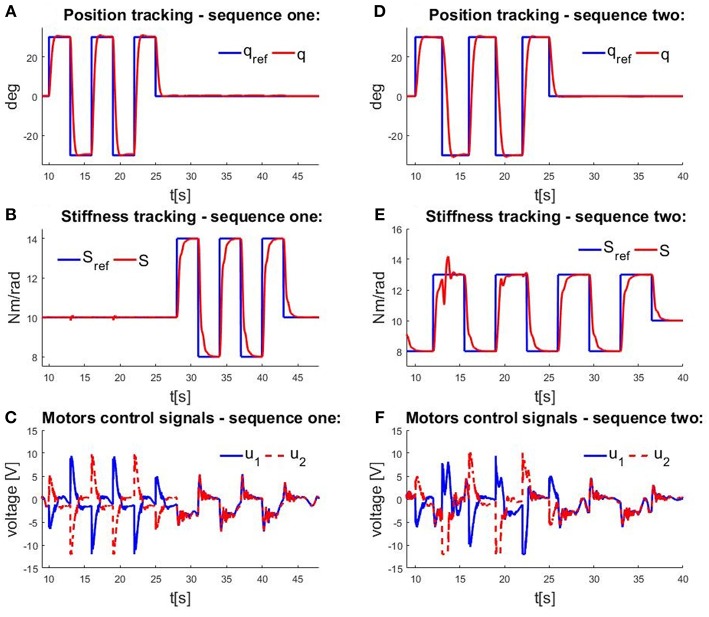
Measurements obtained in experimental testing of a cascade control structure. Time profiles of **(A)** actuator position tracking at constant stiffness; **(B)** actuator stiffness tracking at constant position; **(C)** control signals when position and stiffness are varied individually; **(D)** actuator position tracking when stiffness is varied; **(E)** actuator stiffness tracking when position is varied; and **(F)** control signals when position and stiffness are varied simultaneously.

Figure 7 presents the results of the first experiment. Figures 7A,B show actuator position and stiffness tracking, where the controller was designed for a nominal operating point of *S* = 10.55 Nm/rad. First, the position reference was specified as a step signal sequence in the range from –π/3 to π/3, while stiffness remained at 10 Nm/rad, and then, the position was held constant while stiffness was varied as a pulse signal in the range from 8 to 14 Nm/rad. Figure 7C depicts the control signals of the motors. As expected in the case of actuator position tracking, one motor winds the tendon, while the other unwinds it, as evidenced by the control signals that have the same amplitude with different signs. When stiffness changes, both motors have identical control signal amplitudes because they simultaneously compress and decompress the springs, such that the results are as anticipated. Figures 7D–F show the results when the actuator position and stiffness references were changed consecutively, without time delay, so that both variables were stationary before the reference changes. The results depict position and stiffness tracking, as well as system decoupling, all together with the control effort.

In the second experiment, actuator stiffness tracking is evaluated by measuring tendon forces and actuator position. To test the stiffness, the reference actuator position was set at 0 and the reference stiffness at 10 Nm/rad. In this case, only the stiffness controller is used in the outer loop to achieve compliant behavior. The actuator position control is in the open loop, where regulation relies on the geometrical relation (14) implemented through the static decoupler *D*. Therefore, outer position loop is not aware of the externally applied torque. Trajectory tracking performance will depend on the accuracy of the motor and actuator radii as geometric actuator parameters, which are easy to measure. The torque that acts on the link in the state of equilibrium is equal to 0, such that disturbance *F*_*ext*_, which is a manually entered force that acts tangentially on the actuator link, was measured as the difference between the forces that act on the actuator, measured in the stationary state:

(22) $$\begin{array}{r} F_{ext}=F_{A}-F_{B} \end{array}$$

Instead of a derivative (12), can be represented by means of a linear approximation, as the quotient of the torque change and angle change:

(23) $$\begin{array}{r} S=-\frac{\Delta \phi }{\Delta q} \end{array}$$

Note that externally applied torque is related to externally applied force by a scale factor *r*_*j*_, so actuator stiffness will be estimated from the applied force, which could be directly measured by sensors. Since the nominal angle is 0, and the torque acting on the actuator is *r*_*j*_ (*F*_*A*_ − *F*_*B*_),

(24) $$\begin{array}{r} S=-\frac{r_{j}F_{ext}}{q_{m}} \end{array}$$

holds for (21), where *q*_*m*_ is the instantly measured actuator position (position displacement due to external force *F*_*ext*_). Therefore, the externally applied force could be estimated based on measured actuator position and commanded stiffness as follows:

(25) $$\begin{array}{r} F_{expected}=-\frac{Sq_{m}}{r_{j}} \end{array}$$

**Figure 8:**
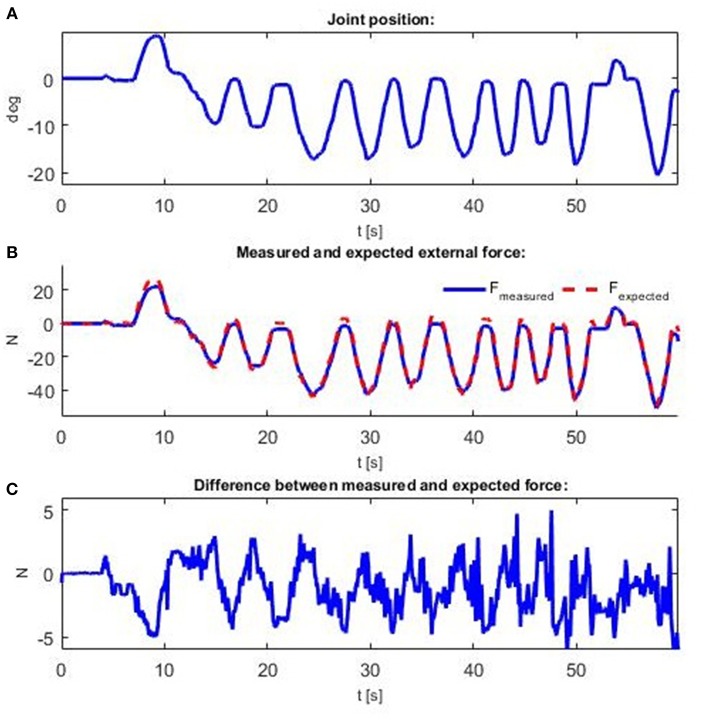
Measurements obtained when an external force is applied. Force is applied manually to create displacement from the actuator's nominal position. Time profiles of: **(A)** actuator position; **(B)** interaction force *F*_*ext*_ and expected force *F*_*expected*_; and **(C)** difference between interaction force *F*_*ext*_ and expected force *F*_*expected*_.

Given that the actuator radius *r*_*j*_ is constant and that the stiffness is held constant, the correlation between the force and the position change is also constant. Figure 8A shows measured actuator positions *q*. The external force *F*_*ext*_, which is the difference between measured forces *F*_*A*_ and *F*_*B*_, and the expected force *F*_*expected*_ based on (23) for the measured position and commanded stiffness are depicted on Figure 8B. It is apparent that the shape of all the graphics is the same and that there is a high level of correlation, but that there are minor deviations between measured and expected forces, as shown in Figure 8C. The amplitude of the differences is relatively small. The differences occur because (22) and (23) are only linear approximations and the stiffness expression is derived according to (13), which is based on spring parameter approximation. The computed root mean square error (RMSE) for the signal from Figure 8C is 2.239 N. This may seem like a relatively high value, but considering that used force sensors have a time delay (10–15 ms), and expected force is computed based on encoder measurements that do not have a time delay, these two signals are not aligned and therefore measured errors and obtained RMSE are presented larger than they really are. Similarly, the two springs are not exactly identical and that introduces additional errors into the modeled system. This experiment demonstrates the practical evaluation of the antagonist VSA stiffness tracking.

## Conclusions

Looking toward future robot actuators that comprise intrinsic compliance for safety and energy efficiency, tendon-driven actuation for reducing robot inertia, and antagonistic configuration with non-linear transmission for enabling bidirectional moves and variable actuator stiffness/compliance, this paper elaborates design and control of tendon-driven compliant antagonistic VSA that can be applied on all antagonistic VSAs. Since the antagonistic design of VSA, mastered by nature through evolution, presents a foundation for moving of humans and mammals in general, this is undoubtedly a design of future robot actuators, which aims to work in the human-centered environment. This research introduces a conventional engineering control approach based on cascading structure to a bioinspired compliant antagonistic actuator.

A cascade structure for simultaneous position and stiffness control of an antagonistic VSA was presented and experimentally tested in the paper. A cascade structure contributed to the stability of the system because a proper inner-loop controller design reduced oscillations at the output from the system, which can occur especially in mechanical systems that feature elastic transmission. Robust controllers were designed for motor positions on the basis of experimental identification of the gearbox motor transfer function. In addition, locally linearized actuator position and stiffness transfer functions were identified with motor position inner-closed loops, such that the controller dynamic was included in the identification. The linear system control theory for controller tuning was applied. A static decoupler was designed based on an estimated spring model and a mechanical design of the actuator.

The advantage of this approach involving system identification is that it requires no previous knowledge about the model of the system and no information about system parameters, such as motor and actuator friction and inertia, gearbox efficiency, force sensor mass, etc. Another advantage is that it is not necessary to measure or control motor torques. Control is achieved via voltage, and the connection between voltage and torque is included in the dynamic that is being identified. Only a part of the system model is required, related to actuator stiffness and position (13) and (14), as well as (15), whose relations were determined experimentally. Consequently, a similar control system design principle can be applied to different types of antagonistic VSAs.

The structure of the I-PD/I-P controllers that were used is selected to satisfy pulling constraint of tendon-driven actuators. The controllers were designed with the desired robustness, such that apart from the dynamics disregarded in the identification process due to their minor effect, and in the case of non-modeled dynamics occurring during continuous operation of the system due to a change in any of the system parameters, the controllers will maintain stability and good trajectory tracking performance. The main feature of the applied controllers is the easy tuning of the system performances as a trade-off between robustness and speed/performance, by changing only one parameter—λ. Therefore, by changing λ online, the great flexibility for the system adaptation to different scenarios and change of the robustness/performances for different operating points are feasibly and easy. The robustness is of great importance in tendon-driven compliant systems so the pulling constraint is successfully tackled.

In order to control antagonistic VSAs more appropriately, especially when a broader stiffness range is available, the controller bank is used. Each controller in the bank is tasked with covering a span around the nominal point for which it was designed, while bumpless switching between controllers is exploited.

Performances of controllers are verified in two experiments: simultaneous position and stiffness tracking while their references are changing separately while actuator stiffness is estimated from the model, and actuator stiffness/compliance control validated by stiffness calculated from force measurements directly.

Extending this control structure to the other types of VSAs requires modification of the current approach. In general, a VSA is a two-input–two-output system. If the actuator stiffness and position are not coupled, the actuator can be observed as two single-input–single-output systems that can be adjusted independently. In a more complex case, if the actuator stiffness and position are coupled, a modification of the proposed approach is needed. The first difference to the presented approach is the identification procedure since input/output dynamic behavior is not symmetric, as it is the case of the antagonistic structure. The same cascade control structure can be kept, but the identification must be conducted from both input channels. To enable the generalization approach presented in this paper to the wider class of the VSAs, it is necessary to overcome the problem of the asymmetric input–output transfer function of VSAs. This can be achieved by introducing the dynamic decoupler that will make outer loop control decoupled from the system. Dynamic decoupler is computed based on locally identified transfer functions (Nordfeldt and Hägglund, 2006), for both input–output channels. For this case, outer loop controllers must be tuned according to the dynamic decoupler values. Now, a new problem arises, dynamic decoupler is locally valid because it is computed according to the locally identified transfer functions. The goal of this paper was to find a simple and yet efficient method for controlling antagonistic actuator as bioinspired VSA; thus, the generalization to the wider class of VSA control will be done in future work, with a focus on how to project robust controllers with a bank of dynamic decouplers.

Further research will examine additional system identification methods, which could lead to more flexible actuator identification procedures and therefore facilitate the broader use of the presented approach to a wider class of VSAs. Also, the influence of different non-linear transmission elements will be evaluated. To increase robustness of the actuator, different approaches to disturbance rejection would be considered (Guo et al., 2018; Yuan et al., 2019). Moreover, the upgrade of the presented results will take into account the inherent asymmetry of the system because the two motors and two springs are not exactly identical. In that case, the elastic elements of arbitrary characteristics can be modeled as higher-order polynomials, where it is not possible to analytically implement a decoupler. Instead, a non-conventional mapping approach would be needed, such as that based on neural networks and initially validated by the authors in Lukic et al. (2018).

## Data Availability

The datasets generated for this study are available on request to the corresponding author.

## Author Contributions

All authors listed have made a substantial, direct and intellectual contribution to the work, and approved it for publication.

### Conflict of Interest Statement

The authors declare that the research was conducted in the absence of any commercial or financial relationships that could be construed as a potential conflict of interest.
