# Supplementary material for: Cascade Control of Antagonistic VSA—An Engineering Control Approach to a Bioinspired Robot Actuator
Source: Front Neurorobot. 2019 Sep 4;13:69. doi: 10.3389/fnbot.2019.00069 (PMC6738013; doi:10.3389/fnbot.2019.00069)
Supplement: Supplementary file 1 [file Data_Sheet_1.docx]

# Appendix A

Closed-loop control structure given in Fig. 9 consists of the plant model $G_{p}\left( s \right)$, controller $C\left( s \right)$, reference $r(t)$, output $y(t)$ and disturbance $d(t)$. Complex controller structure is tuned to reject disturbance. Without of lack of generality, we can consider $r\left( t \right)=0$. Transfer function that describes the influence of disturbance $d\left( t \right)$ to output $y(t)$ as:

$$\frac{Y\left( s \right)}{D\left( s \right)}=\frac{G_{p}\left( s \right)}{1+C\left( s \right)G_{p}\left( s \right)} (24)$$

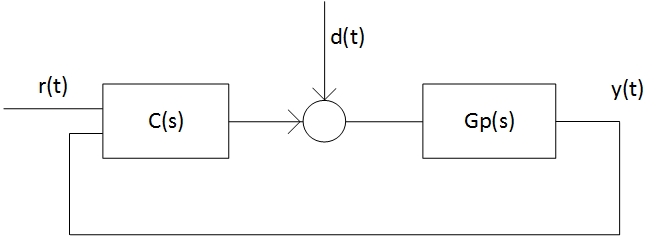


Figure 9. Close-loop control structure.

where $Y\left( s \right)$ and $D\left( s \right)$ are Laplace transformations of disturbance $d\left( t \right)$ to output $y(t)$, respectively.

Equation (24) can be rewritten as:

$$Y\left( s \right)=\left( 1-T\left( s \right) \right)G_{p}\left( s \right)D\left( s \right) \left( 25 \right)$$

where complementary sensitivity function $T\left( s \right)$ is:

$$T\left( s \right)=\frac{C\left( s \right)G_{p}\left( s \right)}{1+C\left( s \right)G_{p}\left( s \right)} \left( 26 \right)$$

Now we want to shape output $Y\left( s \right)$ from eq. (25) with the desired complementary sensitivity function $T_{d}\left( s \right)$ defined as

$$T_{d}\left( s \right)=\frac{\eta\left( s \right)}{\lambda\left( s \right)} \left( 27 \right)$$

$$\eta\left( s \right)= 1+\sum_{i=1}^{n} \eta_{i}s^{i} \left( 28 \right)$$

$$\lambda\left( s \right)= \left( \lambda s+1 \right)^{2n} \left( 29 \right)$$

where $n$ is plant $G_{p}\left( s \right)$ numerator polynomial order. Polynomials $\eta\left( s \right)$ is $n$-th order and $\lambda\left( s \right)$ is
2$n$-th order. These order of polynomials is required to provide causality in controller design.

For desired complementary sensitivity function $T_{d}\left( s \right)$, the desired output function $Y_{d}\left( s \right)$ which represent the response to the disturbance $D\left( s \right)$ is obtained as:

$$Y_{d}\left( s \right)=\left( \frac{\lambda\left( s \right)-\eta\left( s \right)}{\lambda\left( s \right)} \right)G_{p}\left( s \right)D\left( s \right) \left( 30 \right)$$

Equalizing the right sides in eq. (26) and (27) expression for controller $C\left( s \right)$ is obtained as:

$$C\left( s \right)=\frac{\eta\left( s \right)}{\lambda\left( s \right)-\eta\left( s \right)} \frac{1}{G_{p}\left( s \right)} \left( 31 \right)$$

The disturbance is observed as the worst-case scenario as a step signal, thus $D\left( s \right)=1/s$. For the desired $\lambda$ (user-defined to close loop system achieve desired dynamical behavior and/or robustness), parameters of $\eta\left( s \right)$ polynomic are tuned to cancel poles introduced by the system transfer function $G_{p}\left( s \right)$ and disturbance $D\left( s \right)$ in equation (30).

For cases that are in this paper:

- Motor position/voltage is a second-order function thus $n=2$, and $G_{p}\left( s \right)=K_{m}/s(T_{m}s+1)$.
- Actuator stiffness and position are identified as dominantly first-order models, thus $n=1$ and $G_{p}\left( s \right)=K_{m}/(T_{m}s+1)$

For first order$G_{p}\left( s \right)$ the desired output function $Y_{d1}\left( s \right)$ and the desired controller $C_{1}\left( s \right)$

$$Y_{d1}\left( s \right)=\left( \frac{\left( \lambda s+1 \right)^{2}-\left( 1+\eta s \right)}{\left( \lambda s+1 \right)^{2}} \right)\frac{K_{m}}{T_{m}s+1}\frac{1}{s} \left( 32 \right)$$

$$C_{1}\left( s \right)=\frac{\left( 1+\eta s \right)}{\left( \lambda s+1 \right)^{2}-\left( 1+\eta s \right)} \frac{T_{m}s+1}{K_{m}} \left( 33 \right)$$

and for second-order $G_{p}\left( s \right)$ the desired output function $Y_{d2}\left( s \right)$ and the desired controller $C_{2}\left( s \right)$

$$Y_{d2}\left( s \right)=\left( \frac{\left( \lambda s+1 \right)^{4}-\left( 1+\eta_{1}s+\eta_{2}s^{2} \right)}{\left( \lambda s+1 \right)^{4}} \right)\frac{K_{m}}{s\left( T_{m}s+1 \right)}\frac{1}{s} \left( 34 \right)$$

$$C_{2}\left( s \right)=\frac{\left( 1+\eta_{1}s+\eta_{2}s^{2} \right)}{\left( \lambda s+1 \right)^{4}-\left( 1+\eta_{1}s+\eta_{2}s^{2} \right)} \frac{s(T_{m}s+1)}{K_{m}} \left( 35 \right)$$

By substituting the values for the $\eta$(s) polynomial that satisfies eq. (30) pole cancelation, from equations (33) and (35) parameters of controller presented in Table 2 are obtained. Equations (33) and (35) are transfer function form of controllers parameters.
